# Positive Selection on Mammalian Immune Genes—Effects of Gene Function and Selective Constraint

**DOI:** 10.1093/molbev/msaf016

**Published:** 2025-01-21

**Authors:** Mridula Nandakumar, Max Lundberg, Fredric Carlsson, Lars Råberg

**Affiliations:** Department of Biology, Lund University, Lund 223 62, Sweden; Department of Biology, Lund University, Lund 223 62, Sweden; Department of Biology, Lund University, Lund 223 62, Sweden; Department of Biology, Lund University, Lund 223 62, Sweden

**Keywords:** coevolution, molecular evolution, pathogen-mediated selection, pN/pS, Rodentia, gene expression tissue specificity

## Abstract

Genome-wide analyses of various taxa have repeatedly shown that immune genes are important targets of positive selection. However, little is known about what factors determine which immune genes are under positive selection. To address this question, we here focus on the mammalian immune system and investigate the importance of gene function and other factors such as gene expression, protein–protein interactions, and overall selective constraint as determinants of positive selection. We compiled a list of >1,100 immune genes that were divided into six functional categories and analyzed using data from rodents. Genes encoding proteins that are in direct interactions with pathogens, such as pattern recognition receptors (PRRs), are often expected to be key targets of positive selection. We found that categories containing cytokines, cytokine receptors, and other cell surface proteins involved in, for example, cell–cell interactions were at least as important targets as PRRs, with three times higher rate of positive selection than nonimmune genes. The higher rate of positive selection on cytokines and cell surface proteins was partly an effect of these categories having lower selective constraint. Nonetheless, cytokines had a higher rate of positive selection than nonimmune genes even at a given level of selective constraint, indicating that gene function per se can also be a determinant of positive selection. These results have broad implications for understanding the causes of positive selection on immune genes, specifically the relative importance of host–pathogen coevolution versus other processes.

## Introduction

Studies of a wide diversity of taxa have consistently shown that immune genes and other genes at the host–pathogen interface are among the most important targets of positive selection, i.e. selection driving divergence between species (Sackton et al. 2007; Kosiol et al. 2008; Enard et al. 2016; Shultz and Sackton 2019; Roycroft et al. 2021). Moreover, several studies have found that positive selection primarily targets certain immune pathways or functional categories of immune genes. A seminal study of *Drosophila* found that signatures of positive selection are enriched in genes encoding receptors involved in recognition of pathogens—so-called pattern recognition receptors (PRRs)—but not in immune genes encoding signal transduction proteins or effectors (Sackton et al. 2007; see also e.g. Kosiol et al. 2008; Obbard et al. 2009; Shultz and Sackton 2019). An emerging picture is that genes encoding proteins involved in direct physical interactions with pathogens—either because the function of the host protein is to target some pathogen molecule or because pathogens target the host protein to infect or manipulate host immunity—are particularly important targets of positive selection (Cagliani et al. 2016; Enard et al. 2016; Shultz and Sackton 2019; Souilmi et al. 2021). This pattern is consistent with the idea that positive selection is driven by reciprocal adaptation between hosts and pathogens, leading to antagonistic coevolution (Sackton et al. 2007; Dybdahl et al. 2014).

**Fig. 1.**
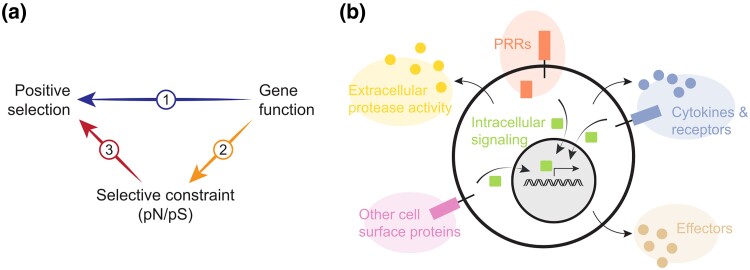
a) Differences in rates of positive selection between functional categories of immune genes could be a direct effect of their function (arrow 1) or an indirect effect of differences in selective constraint (arrows 2 and 3). b) Immune genes were divided into six different categories depending on the function of the encoded protein: (1) PRRs recognize “pathogen-associated molecular patterns” (PAMPs; i.e. molecular structures unique for microbes). Sensors of “damage-associated molecular patterns” (DAMPs, i.e. endogenous molecules reflecting tissue damage) were not included. (2) Cytokines, chemokines, and their receptors, also including anaphylatoxin receptors. (3) Other cell surface proteins include receptors and ligands involved in cell–cell interactions (e.g. CD80–CD28, Fas–FasL), and Fc and complement receptors. (4) Intracellular signaling proteins are involved in intracellular signal transduction and include adaptors, enzymes such as kinases, ubiquitinases, and proteases, transcription factors and cofactors, etc. (5) Extracellular protease activity includes secreted proteases and protease inhibitors, mainly involved in the complement and coagulation pathways. (6) Effectors are proteins involved in killing of pathogens by direct interaction, including antimicrobial peptides, restriction factors, and various other proteins.

Besides gene function, the degree of selective constraint can also determine whether a gene is under positive selection, as well as the power to detect selection (Enard et al. 2016). Genes that have low expression level, tissue-specific expression, long coding sequence (CDS), or encode proteins involved in few interactions with other proteins are generally less constrained than other genes (Zhang and Yang 2015; Soni and Eyre-Walker 2022). Immune gene categories found to be important targets of selection, for example, genes encoding PRRs, might have relatively low and tissue-specific expression and few protein–protein interactions (PPI). Thus, enrichment of signatures of selection in PRR genes or other functional categories of immune genes could potentially be an indirect effect of genes in these categories being less constrained, rather than that genes in certain categories are particularly important targets of selection because of their function per se (Fig. 1a).

Here, we focus on the mammalian immune system to determine which functional categories of immune genes are key targets of positive selection and whether enrichment of signatures of positive selection in certain categories is a direct effect of gene function or an indirect effect of other factors. To this end, we compiled a list of >1,100 immune genes and divided them into six functional categories depending on the function of the encoded protein. Using sequence and expression data from rodents, we then tested which of these functional groups of immune genes have higher rates of positive selection compared with a set of nonimmune control genes. We also tested whether any differences between categories remained when controlling for selective constraint, either by using a direct measure of selective constraint (pN/pS; Schloissnig et al. 2013), or various potential determinants of selective constraint (mean expression level, tissue specificity of expression, number of PPI, and gene length) as covariates in the statistical models.

## Results

To analyze selection on mammalian immune genes, we focused on two rodent families, Cricetidae and Muridae, within the superfamily Muroidea. These two families are the most species-rich among rodents, following rapid radiation to multiple habitats across all continents around 25 million years ago (Fabre et al. 2012). This, together with comprehensive genomic resources for the house mouse (*Mus musculus*), makes them uniquely suited for analyses of positive selection in mammals. We used publicly available genomes and annotations from 30 species (15 species from each family; supplementary fig. S1, Supplementary Material online).

### Immune Gene Categories

To compile a list of mammalian immune genes, we obtained information about genes of the immune system or similar processes for the house mouse from Amigo2 (Aleksander et al. 2023), KEGG (Kanehisa et al. 2017), Panther (Mi et al. 2019), and Reactome (Fabregat et al. 2018). The overlap between these four sources was relatively low (supplementary fig. S2, Supplementary Material online). We therefore selected genes that were classified to have immunological function by at least two sources, plus a few well-established immune genes that were only listed by one source (e.g. some defensins and restriction factors). We excluded all major histocompatibility (MHC), T-cell receptor, and immunoglobulin heavy- and light-chain genes, as they are gene families with extensive duplications and tend to be poorly annotated in most genome assemblies of nonmodel organisms. This resulted in a list of 1,273 immune genes (supplementary table S1, Supplementary Material online). Of these, 1,112 could be divided into six functional categories (Fig. 1b, Table 1; supplementary table S1, Supplementary Material online).

**Table 1:** Number of house mouse genes originally considered for each gene category and number of genes in each category eventually included in different analyses

Gene category^a^	No. in original list of genes	No. of alignments w sequence data from ≥7 species in each family	No. included in analyses w bank vole pN/pS data
PRR	74	56	51
Cyto/chemokines and their receptors (cytokines)	211	179	161
Other cell surface proteins (cell surface proteins)	229	144	129
Intracellular signaling proteins (signaling proteins)	425	400	333
Extracellular protease activity (proteases)	53	36	29
Effectors	120	47	34
Uncategorized immune genes	161	115^b^	NA
Nonimmune control genes	1,300	1,005	859

^a^Abbreviated category names used in main text and figures in parentheses.

^b^Only included in comparison with all immune versus nonimmune control genes.

The goal of the division was to distinguish immune genes encoding proteins which by their function are involved in direct interactions with pathogens (i.e. PRRs and effectors) from other immune genes. This is because host–pathogen coevolution is generally considered to involve host and pathogen genes whose products interact physically (Dybdahl et al. 2014). Thus, categories where all genes encode such proteins (PRRs and effectors) could be expected to be key targets of positive selection as a direct effect of their function. The categorization is inspired by previous work dividing *Drosophila* immune genes into recognition, signaling, and effectors (Sackton et al. 2007), and mammalian immune genes into sensors, cytokines, effectors, etc. (Deschamps et al. 2016; Hagai et al. 2018).

To obtain a set of control genes, we randomly selected 1,300 protein-coding mouse genes that are not associated with immune-related processes (supplementary table S1, Supplementary Material online).

### Tests for Positive Selection

We performed tests for positive selection based on calculating dN/dS (or *ω*), the ratio of nonsynonymous and synonymous substitution rates. Values of dN/dS < 1, =1 or >1 indicate purifying selection (selective constraint), neutral evolution, and positive selection, respectively. When calculated for a whole CDS, dN/dS reflects the combined effect of positive and purifying selection and is rarely >1. To test for positive selection, we therefore used codon models implemented in BUSTED (Murrell et al. 2015), part of the HyPhy package (Kosakovsky Pond et al. 2020). BUSTED tests for the presence of a nonzero proportion of codons with dN/dS > 1 in at least one lineage in an alignment (Murrell et al. 2012). Recent studies have shown that variation in the rate of synonymous substitutions among sites (S) and the presence of multinucleotide substitutions (or multihit substitutions; MH) can lead to false positives in tests for positive selection (Venkat et al. 2018; Wisotsky et al. 2021; Lucaci et al. 2023). For each alignment, we therefore specified three different models in BUSTED: without S or MH, with S, and with S and MH. We then used model-averaging to synthesize the results from the three models, as recommended by Lucaci et al. (2023). To benchmark our selection analyses against previous studies of positive selection on immune genes (performed before the potential importance of S and MH was widely acknowledged), we also report results from BUSTED without S and MH. In addition, we tested for positive selection analyses using another popular program for selection analysis, CODEML in the PAML package (Yang 2007), which does not allow S and MH. Here, we compared the models M1a versus M2a, which tests for the presence of codons with dN/dS >1 across the whole phylogeny (Álvarez-Carretero et al. 2023).

As in previous studies (Venkat et al. 2018; Wisotsky et al. 2021; Lucaci et al. 2023), accounting for S and MH drastically reduced the proportion of genes with signatures of positive selection (Table 2). Regardless of model, the rate of positive selection was 1.5 to 2.3 times higher for immune than control genes. In the analyses below, we focus on the results from BUSTED with model-averaging and FDR correction, which represents the most conservative approach. The immune genes thus identified as having signatures of positive selection include several that are well known, such as the PRRs *Ifih1* and *Tlr1,* the cytokines *Ifnb1* and *Il12b,* the cytokine receptors *Ifngr2* and *Il12br1,* and the cell surface protein *Cd80* (supplementary table S2, Supplementary Material online).

**Table 2:** Number of control and immune genes with signatures of positive selection as determined by different models, at nominal *P* value (*P* < 0.05) and with FDR correction at *q* < 0.2

Model	N control genes (of 1,005)	*N* immune genes (of 977)	Likelihood ratio test (df = 1)
PAML M1a versus M2a	142 (14.1%)	307 (31.4%)	*χ* ^2^ = 86.0, *P* < 0.0001
PAML M1a versus M2a *q* < 0.2	138 (13.7%)	305 (31.2%)	*χ* ^2^ = 88.9, *P* < 0.0001
BUSTED (−S −MH)	201 (20.0%)	356 (36.4%)	*χ* ^2^ = 66.9, *P* < 0.0001
BUSTED (−S −MH) *q* < 0.2	218 (21. 7%)	363 (37.2%)	*χ* ^2^ = 57.6, *P* < 0.0001
BUSTED model-average	89 (8.9%)	127 (13.0%)	*χ* ^2^ = 8.79, *P* = 0.0030
BUSTED model-average *q* < 0.2	20 (2%)	37 (3.8%)	*χ* ^2^ = 5.80, *P* = 0.016

BUSTED (-S -MH) represents results from a model that does not take variation in rate of synonymous substitutions (S) and multinucleotide substitutions (MH) into account. BUSTED model-average reports a synthesis of three models with and without synonymous rate variation and multinucleotide substitutions.

### Selection on Different Categories of Immune Genes

**Fig. 2.**
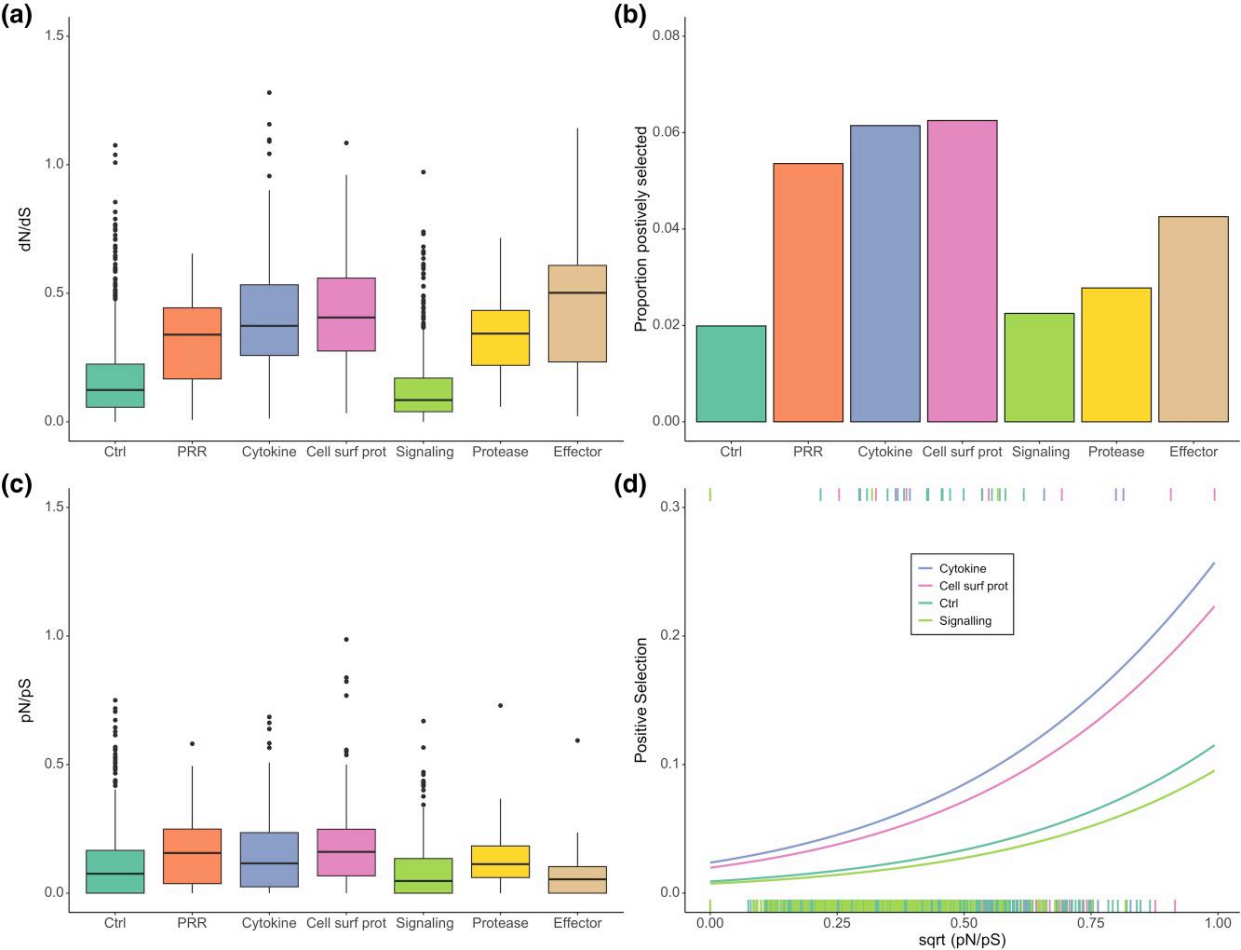
Selection on different categories of immune genes and nonimmune control genes. a) Box plot of dN/dS. b) The proportion of genes with signatures of positive selection. c) Box plot of pN/pS. d) The proportion of genes with signatures of positive selection against pN/pS for three of the categories of immune genes and nonimmune control genes. Lines are predictions from a binomial general linear model with positive selection against gene function and pN/pS.

We first compared the overall mode of selection on the full protein CDS of different categories of genes. We found a significant difference in dN/dS between categories (*F*_6, 1860_ = 110.8, *P* < 0.0001; Fig. 2a). All immune gene categories had substantially higher dN/dS than control genes (Dunnett's test: all *P* < 0.0001) except for intracellular signaling proteins, which had significantly lower dN/dS than controls (Dunnett's test: *P* = 0.0005).

To investigate whether the higher dN/dS of some categories of immune genes as compared to nonimmune control genes was driven by positive selection on a subset of codons, we compared the proportions of genes with signatures of positive selection, as determined by BUSTED with model-averaging and FDR correction (see above). Like dN/dS, the proportion of genes with signatures of positive selection differed between gene categories (*χ*^2^ = 14.6, df = 6, *P* = 0.023; Fig. 2b), although only the categories cytokines and cell surface proteins had a significantly higher proportion of genes with signatures of positive selection than the controls (Dunnett's test: *P* = 0.014 and 0.023, respectively). PRR genes had a similar rate of positive selection as genes in the categories cytokines and cell surface proteins but did not differ significantly from controls due to a lower number of genes in the PRR category (56 genes, of which three were under positive selection). It should also be noted that the categories effectors and extracellular proteases contained <50 genes each, of which only 1 to 2 had signatures of positive selection. The estimates of the rate of positive selection on genes in these categories should thus be interpreted with caution.

### Selection on Different Categories of Immune Genes While Controlling for Selective Constraint

The higher rate of positive selection on cytokines and cell surface proteins could be a direct effect of their function. Alternatively, these categories could have lower selective constraint, which in turn might affect the rate of positive selection (Fig. 1a). To disentangle these scenarios, we first compared the extent of selective constraint of different categories and then performed analyses where we compared the rate of positive selection on different categories while controlling for selective constraint.

We used pN/pS—the ratio of the rates of nonsynonymous and synonymous polymorphisms—as a direct measure of selective constraint, where a lower value means higher selective constraint (Schloissnig et al. 2013; see Enard et al. 2016 for a discussion of the utility of pN/pS as a measure of selective constraint). To estimate pN/pS, we used population genomic data from one species in each family: the house mouse (*Mus musculus*; Muridae) and the bank vole (*Myodes glareolus*; Cricetidae). pN/pS for house mouse and bank vole orthologs was moderately correlated (*r*_S_ = 0.32, *P* < 0.0001, *N* = 1,693 immune and control genes). We used mean pN/pS across house mouse and bank vole orthologs in the analyses below, to make it as representative as possible for the whole phylogeny. The bank vole reference genome is more fragmented and less comprehensively annotated than the house mouse genome. Consequently, there were fewer genes available for analyses involving bank vole pN/pS data (Table 1).

There was a highly significant difference in pN/pS between gene categories (Kruskal–Wallis test: *χ*^2^ = 77.9, df = 6, *P* < 0.0001; Fig. 2c). PRRs, cytokines, and cell surface proteins had higher pN/pS than controls (Dunn's: *P* ≤ 0.013), while signaling proteins had lower pN/pS (Dunn's: *P* = 0.0029). Thus, differences in pN/pS between categories largely matched differences in rates of positive selection (Fig. 2b), suggesting that there might be an indirect effect of selective constraint on positive selection (Fig. 1a). All immune gene categories except intracellular signaling proteins had higher dN/dS than nonimmune control genes even when controlling for pN/pS (supplementary fig. S3 and supplementary table S3, Supplementary Material online).

To test whether the difference between gene categories in rates of positive selection (Fig. 2b) was confounded by pN/pS, we performed a general linear model with gene category as factor and pN/pS as covariate. Given the overall low rate of positive selection and the reduction in sample size due to missing values for pN/pS, we only included the immune gene categories that contained ≥100 genes in this analysis to improve statistical power, that is, cytokines, cell surface proteins, and signaling proteins. We first confirmed that the effect of gene function remained in this subset (*χ*^2^ = 13.4, df = 3, *P* = 0.0038; Dunnett's: *P* = 0.015 and *P* = 0.027 for cytokines and cell surface proteins, respectively). The rate of positive selection showed a positive relationship with pN/pS (*χ*^2^ = 12.5, df = 1, *P* = 0.00040; Fig. 2d). Including pN/pS as a covariate in the model reduced the effect of the factor gene category (*χ*^2^ = 7.89, df = 3, *P* = 0.048), and only cytokines now showed a higher rate of positive selection than controls (Dunnett's: cytokines, *P* = 0.048; cell surface proteins, *P* = 0.21). Thus, the higher rate of positive selection in cytokines and cell surface proteins seems to be partly an indirect effect of these categories having lower selective constraint. Nevertheless, cytokines still had a higher rate of positive selection than controls even when controlling for pN/pS, indicating that there is also a direct effect of gene function at least for this category.

Using only house mouse pN/pS, and hence a larger number of genes (Table 1) but an estimate of selective constraint that is less representative for the whole phylogeny, yielded identical conclusions (model without pN/pS as covariate: gene category: *χ*^2^ = 13.4, df = 3, *P* = 0.0039; Dunnett's: cytokines, *P* = 0.0067; cell surface proteins, *P* = 0.011; model with pN/pS as covariate: gene category: *χ*^2^ = 10.9, df = 3, *P* = 0.012; pN/pS: *χ*^2^ = 11.4, df = 1, *P* = 0.00074; Dunnett's: cytokines, *P* = 0.0068; cell surface proteins, *P* = 0.057; note also the slightly weaker effect of pN/pS in this model compared with the model above where pN/pS was based on data from two species).

Using tests that do not take into account variation in the rate of synonymous substitutions among sites and the presence of multinucleotide substitutions (Lucaci et al. 2023) also yielded similar results. However, more of the immune gene categories showed a higher rate of positive selection than nonimmune control genes, even when controlling for selective constraint (all immune gene categories except signaling proteins when analyses were based on results from the least conservative tests for positive selection).

### Determinants of Selective Constraint

To investigate what aspect(s) of selective constraint might influence positive selection, we obtained data on potential determinants of selective constraint, including mean gene expression (across tissues), tissue specificity of expression, number of PPI, and gene sequence length.

We used gene expression data for ten organs from house mouse and bank vole. For house mouse, we used data from the ENCODE project (Dunham et al. 2012), with 1 to 4 samples per organ. For the bank vole, we performed RNA sequencing of 3 to 18 samples per organ from both males and females collected during both summer and winter. To measure tissue specificity of expression for each gene, we calculated *τ*, an index reflecting breadth of expression, where a higher value means more tissue-specific expression (Yanai et al. 2005). As expected from previous studies (Brawand et al. 2011), samples grouped by organ rather than species in a PCA (supplementary fig. S4, Supplementary Material online). Additionally, mean expression and tissue specificity of house mouse and bank vole orthologs were strongly correlated (*r*_S_ = 0.78 and 0.73, respectively; supplementary fig. S5, Supplementary Material online). These results indicate that expression patterns are highly conserved across these two rodent families. Data on the number of PPI for house mouse were obtained from the STRING database (Szklarczyk et al. 2023). The different potential determinants of selective constraint were weakly–moderately correlated with each other (−0.31 < *r*_S_ < 0.24; supplementary fig. S6, Supplementary Material online).

pN/pS was independently correlated with all the measured potential determinants of selective constraint, although relatively weakly so (partial Spearman’s rank correlations: mean expression: *r*_S_ = −0.15; *τ*: *r*_S_ = 0.09; PPI: *r*_S_ = −0.095; sequence length: *r*_S_ = 0.17; all *P* < 0.0002). Thus, all these gene properties were associated with selective constraint, though the considerable residual variation in pN/pS indicates there must be additional important determinants of selective constraint. All the determinants of selective constraint were independently associated with dN/dS (supplementary fig. S3 and supplementary table S4, Supplementary Material online), indicating that these factors affected the overall mode of selection. Moreover, all immune gene categories except intracellular signaling proteins had higher dN/dS even when including the determinants as covariates (Dunnett's: *P* < 0.009).

To test whether mean expression, *τ*, PPI, and/or sequence length contributed to the indirect effect of selective constraint on positive selection (Fig. 2d), we performed a general linear model with these variables as covariates and gene category as factor. None of the covariates were significant predictors of positive selection (*P* > 0.08). Thus, the differences in the rate of positive selection between gene categories could not be explained by any of the determinants of selective constraint considered here.

## Discussion

Our analyses showed that two functional categories of immune genes—cytokines and cell surface proteins—had a significantly higher proportion of genes with signatures of positive selection than control genes. PRRs had a similar rate of positive selection as cytokines and cell surface proteins, but the relatively low number of PRR genes limited statistical power. The higher rate of positive selection on cytokines and cell surface proteins was partly an indirect effect of lower selective constraint. However, cytokines had a higher rate of positive selection even when controlling for selective constraint, indicating that there is also a direct effect, such that cytokines are important targets of positive selection because of their function per se. We also found that overall selective constraint was influenced by gene expression patterns, PPI, and gene length, but none of these factors explained the effect of selective constraint on positive selection.

During the last decade, analyses of pathogen-mediated selection in animals have broadened the focus from MHC genes to include also other functional categories, particularly PRRs (Vinkler and Albrecht 2009; Vinkler et al. 2023). The reason for focusing on PRRs is that genes encoding proteins involved in direct interactions with pathogens—such as PRRs—are prime candidates for coevolution (Sackton et al. 2007; Dybdahl et al. 2014). The rationale behind this idea is that a change in a molecule of the pathogen to evade recognition by the corresponding PRR should select for counteradaptation in the host, which will renew selection for evasion, and so on. Such coevolution of directly interacting host and pathogen molecules forms the basis of classic theories of host–pathogen coevolution, like “gene-for-gene” models, which readily generate persistent positive selection (Agrawal and Lively 2002; Dybdahl et al. 2014; Ebert and Fields 2020; Råberg 2023; see Adrian et al. (2019) for a concrete example in mammals likely reflecting this process). Positive selection on PRR genes has been found in several vertebrate taxa (Wlasiuk and Nachman 2010; Alcaide and Edwards 2011; Babik et al. 2014; Velová et al. 2018), but our analyses indicate that PRRs are no more important targets of positive selection than many other immune genes; why is that?

As noted by, for example, Wlasiuk and Nachman (2010), the expectation of widespread positive selection in PRR genes is not entirely consistent with the concept of pattern recognition as originally defined by Janeway (1989), where the ligand (PAMP) recognized by each PRR is assumed to be constrained and more or less invariant across microbes, allowing for a limited set of invariant innate receptors (i.e. PRRs) to induce a host response against all types of microbial invasion. Even though there are several examples of evolutionary modification of PAMPs to evade recognition (Murphy et al. 2022, p. 619), meaning that there is indeed scope for PRR–PAMP coevolution in at least some cases, there is also evidence that PRRs may recognize PAMPs in a way that minimizes the possibility for immune evasion. For example, NAIP5 binds multiple surfaces of flagellin so that several simultaneous mutations in flagellin are required to escape recognition (Tehthorey et al. 2017), which should limit the potential for antagonistic coevolution between PRR and PAMP. Thus, the fact that many PAMPs are inherently constrained together with PRR multiple surface binding of PAMPs could explain why PRR genes do not stand out as targets of positive selection in our analyses. Instead, our results suggest that further broadening of the types of genes included in analyses of pathogen-mediated selection would be valuable for a comprehensive understanding of the evolutionary consequences of host–pathogen interactions. Of the gene categories delineated in the present study, cytokines and cytokine receptors appear to be especially relevant (see also Turner et al. 2012).

Why then are cytokines and their surface receptors important targets of positive selection, beyond what can be expected from their level of selective constraint? One reason might be that they are common targets of pathogen immune evasion strategies—where pathogens evolve to interfere with the immune response—or used as receptors by pathogens for infection of host cells. Under both scenarios, one could expect coevolution where the host is evading the pathogen. Indeed, some viruses produce proteins that bind cytokines or cytokine receptors, thereby interfering with these host proteins (Murphy et al. 2022, p. 631). There are, however, also numerous examples of pathogens interfering with proteins in the category intracellular signaling (Murphy et al. 2022, p. 626). In fact, for many pathogens, intracellular signaling proteins appear to be the main targets of immune evasion strategies. For example, of the immune evasion mechanisms of SARS-CoV-2 targeting interferon responses (key cytokines mediating antiviral effects), the vast majority interfere with intracellular signaling proteins (Beyer and Forero 2022). Based on this, genes in the intracellular signaling category would be expected to be key targets of selection. However, our results indicate that such examples serve as exceptions rather than the rule. Even though there are genes in the intracellular signaling category with signatures of positive selection, such genes on average evolve in a similar manner as nonimmune genes, even when controlling for selective constraint (cf. Enard et al. 2016). In this context, it is interesting to note that pathogens have either intracellular or extracellular lifestyles, meaning that they are thought to primarily reside and replicate within or outside of host cells. By necessity, however, intracellular pathogens also have extracellular phases during an infection. The realm for potential host–pathogen interactions is therefore expected to be larger for extracellular compared with intracellular host molecules—a feature that might contribute to our finding that cytokines and their receptors are key targets of positive selection.

A potential additional explanation for positive selection on cytokines—besides the form of host–pathogen coevolution sensu stricto described above (i.e. reciprocal adaptation between a host and pathogen gene)—is consecutive epidemics with different pathogens or repeated spillover of a pathogen from a reservoir host, as in plague (Demeure et al. 2019). Under this scenario, epidemics might lead to repeated episodes of positive selection in the host, but without necessarily leading to counteradaptations in the pathogen. Importantly, such a process does not represent coevolution, and it would seem to be less dependent on direct interactions between a host protein and pathogen molecule. This mode of pathogen-mediated selection—which we call epidemic selection—could potentially explain the positive selection in immune genes that generally do not have direct interactions with pathogens but play a key role in regulating immune responses, such as cytokines.

To conclude, our analyses indicate that cytokines and their receptors as well as cell surface proteins involved in, for example, cell–cell interactions are key targets of positive selection in the immune system. This seems to be partly an indirect effect of low selective constraint. However, at least cytokines and cytokine receptors had a higher rate of positive selection even when controlling for selective constraint, indicating that they are important targets of selection as a direct effect of their function. This could be a result of cytokines and their receptors being common targets of pathogen immune evasion, leading to reciprocal adaptation due to direct host–pathogen interactions (i.e. coevolution). Alternatively, other processes, such as adaptation to consecutive epidemics of different pathogens without pathogen counteradaptation (i.e. “epidemic selection”), may play an important role in driving positive selection on cytokines even if they are not involved in direct interactions with pathogens.

## Materials and Methods

### Selection and Categorization of Immune Genes

To compile a list of immune genes, we obtained information for the house mouse from four sources: Amigo2 (all genes in Immune system process [GO0002376]; *N* = 2270) (Aleksander et al. 2023), KEGG (all genes in Immune system pathways [5.1]; *N* = 1239) (Kanehisa et al. 2017), Panther (all genes in Immune system process; *N* = 999) (Mi et al. 2019), and Reactome (all genes in subcategories under Innate Immune System, Adaptive Immune System, and Cytokine Signaling in Immune System, except Growth Hormone Receptor Signaling and Prolactin Receptor Signaling; *N* = 1,754) (Fabregat et al. 2018). Duplicates and genes with names not recognized by Ensembl BioMart were excluded. Categorization of genes was based on information at UniProt (The UniProt Consortium 2017), KEGG pathway cytokine–cytokine receptor interaction (pathway 04060), AnimalTFDB (Shen et al. 2023), and APD3 (Wang et al. 2016). In a few cases, especially for PRRs and effectors, information from original publications was obtained (supplementary table S1, Supplementary Material online).

It should be noted that the results from the kind of comparison of gene categories performed here are sensitive to the criteria used for categorization. For the vast majority of genes, the categorization is unambiguous, but some cases are debatable, especially as regards PRRs. For example, some of the genes in the complement system have been reported to act as PRRs (*C1qa, C1qb, C1qc, Cfp*), but are considered to have other primary functions (C1q binds to the Fc part of antibodies; Cfp acts as a positive regulator of the alternative pathway by stabilizing the C3bBb complex [Murphy et al. 2022]). To avoid “diluting” the PPR category with genes that primarily have other functions, we chose a restrictive approach and only included genes whose primary function is recognition of PAMPs.

### Species Selection and Genome Annotations

We used publicly available genomes of species within rodent families Cricetidae and Muridae, deposited in the NCBI (supplementary table S5, Supplementary Material online). We limited the species included in the analysis based on two criteria. First, to preserve consistency in gene annotation methods, we selected genomes for which genome annotation was available with the Tool to infer Orthologs from Genome Alignments (TOGA) (Kirilenko et al. 2023). TOGA is a machine-learning-based ortholog identifier that uses annotation information from a reference species to identify orthologous genes in closely related species. Additionally, TOGA can identify orthologous genes split across multiple scaffolds in a fragmented genome, making it ideal to work with draft genomes. TOGA annotations for Cricetidae and Muridae, with house mouse as the reference organism, were downloaded from http://genome.senckenberg.de/download/TOGA/. Second, to prevent overrepresentation of species from a single genus, which could potentially bias selection analyses, we limited the representation from each genus to a maximum of two species. Application of these two filters resulted in 30 genomes for final analysis, with 15 species each from Muridae and Cricetidae, spread across three and four subfamilies, respectively (supplementary fig. S1, Supplementary Material online).

### Sequence Extraction and Multiple Sequence Alignment

For both the immune and control gene set, house mouse transcripts were extracted with the package biomaRt (Durinck et al. 2009) using R 4.3 (R Core Team 2022). We only retained those genes that encoded an active protein, and for these genes, the longest transcript was selected. Using this transcript information, orthologous transcripts in other species were fetched using TOGA, and only those classified as “one-to-one” orthologs in each species and projected to be intact or with “uncertain signals of loss” were retained.

Multiple sequence alignment for each gene was generated using PRANK v.170427 (Löytynoja and Goldman 2008) using a codon substitution model (Kosiol et al. 2007). Each alignment from PRANK was manually checked for issues in Geneious 11 (Biomatters Ltd). Poorly aligned regions, specifically regions with multiple short (one to a few codons) indels, were removed. The trimmed sequences were checked for length, and genes that were <80% of the starting alignment length were excluded. Also, alignments with <7 sequences from each family were excluded. This yielded 977 immune and 1,005 control genes for further analysis.

### Phylogeny Reconstruction

Unrooted gene trees for each alignment were generated using IQ-TREE2 (Minh et al. 2020). The best model for each gene was determined with ModelFinder (Kalyaanamoorthy et al. 2017) with UltraFast Bootstrapping for 1,000 runs (Hoang et al. 2018).

### Positive Selection Analysis

Gene-wide estimates of positive selection were determined using BUSTED from the HyPhy package (Murrell et al. 2015) using gene trees. Genes with signals of positive selection were determined by comparing the likelihood values from the neutral model (*ω* = 1) against the positive selection model where *ω* > 1 is allowed, using a likelihood ratio test. To account for variation in the rate of synonymous substitutions among sites (S) (Wisotsky et al. 2021) and the presence of multinucleotide substitutions (or multihit substitutions; MH) (Venkat et al. 2018), BUSTED was run in three modes across the entire phylogeny following (Lucaci et al. 2023): (i) without S and MH, (ii) with S, and (iii) with S and MH. Significance of positive selection was determined using model-averaged *P*-value as described in Lucaci et al. (2023), which favors the best-fitting model while penalizing poor-fitting models.

To compare the results from BUSTED with other established methods, we also used the program CODEML implemented in PAML v4.10.7 (Yang 2007) for detecting genes under positive selection using gene trees. Models were run with no assumption of molecular clock, and codon frequencies were estimated as average nucleotide frequencies at the three codon positions. As potentially problematic sites were removed manually in the previous step, cleandata = 0 was used. Similar to BUSTED, genes with signals of positive selection were determined by comparing the likelihood values from M1a (neutral model; *ω* constrained to 1) versus M2a (positive selection model; *ω* > 1 allowed) using a likelihood ratio test (Álvarez-Carretero et al. 2023).

Significance values were corrected for multiple testing using the Benjamini–Hochberg method (Benjamini and Hochberg 1995).

### Selective Constraint Within Species

To characterize the selective constraint within rodent species, we used degenotate v. 1.3 (Mirchandani et al. 2024) to calculate pN/pS for the house mouse and the bank vole. For the house mouse, we downloaded a set of quality-filtered variants from natural European mice populations (Harr et al. 2016). This dataset was filtered to contain only German samples (*N* = 8), biallelic SNPs and at least six genotypes without missing data, which resulted in 14.6 million variants. We further downloaded the mm10 (GRCm38.p4) house mouse genome assembly, which had been used when calling variants, and the corresponding gene annotation (gencode.vM10.annotation.gtf) from Gencode (https://www.gencodegenes.org/).

To obtain variant data from the bank vole, trimmed reads from 31 bank voles used in Lundberg et al. (2020) were mapped to the reference genome using bwa mem 0.7.17-r1188 with default settings except for specifying an -M flag for downstream compatibility and adding a read group header (-R flag). The alignments were converted into sorted BAM files using samtools 1.17 (Danecek et al. 2021), and read duplicates were removed using Picard tools 2.10.3 (http://broadinstitute.github.io/picard).

From the filtered alignments, we called variants using FreeBayes 0.7.17-r1188 (Garrison and Marth 2012). To speed up the analysis, we used GNU Parallel 20180822 (Tange 2022) to run FreeBayes in parallel across scaffolds. We further restricted the variant calling to consider a maximum of four alleles (−use-best-n-alleles 4) and to only include intervals without excessive coverage. To obtain intervals of excessive coverage, we used samtools depth with all the bam files as input and calculated, for each site, the total coverage across all samples. Next, we calculated the mean total coverage across all sites (2,766) and used bedtools 2.29.2 (Quinlan and Hall 2010) to merge adjacent sites where the total coverage was at least twice the mean total coverage into excessive coverage intervals. These intervals had a combined length of 2.27 Gb or 97.5% of the total assembly.

The raw set of variants was filtered with vcflib 1.0.1 (Garrison et al. 2022) to only contain variants with a quality higher than 30 (QUAL > 30) and alternative alleles that were supported by at least one read on each strand (SAF > 0 and SAR > 0) and at least two reads centered to the left and the right (RPL > 1 and RPL > 1). Multinucleotide polymorphisms and complex alleles (composites of different allele types) were decomposed into SNPs and indels.

Next, we filtered variants based on coverage using vcftools 0.1.16 (Danecek et al. 2011). As both males and females were included among the resequenced samples, we first partitioned the assembly into a set of autosomal and X-linked scaffolds. To this end, we used the multicov function in bedtools to count reads in 1-kb windows for six males and six females in the dataset. We obtained normalized read counts for each sample by dividing the raw window-based read counts with the median read count across all windows. For each window, we calculated the mean normalized coverage for males and females and plotted the results in R. Using a manual inspection of the plots, we assigned 60 scaffolds (in total 140 Mb) with on average 2:1 female–male coverage to the X chromosome. For the autosomal scaffolds, we identified, for each sample, sites where the coverage was below one third or higher than 1.5 times the sample-specific median. For X-linked scaffolds, the same coverage thresholds were used for females, and for males, the thresholds were halved. The sites from all samples were combined together with monomorphic sites and removed from the dataset.

In the following steps, we filtered the data to only contain biallelic SNPs that had an allele balance (fraction of reads supporting the reference allele in heterozygotes) between 0.3 and 0.7, a Hardy–Weinberg excess heterozygote *P* value higher than 0.0001 and that had fewer than 5/31 missing genotypes.

Finally, we removed SNPs that overlapped with annotated repeats. Repeats were de novo identified in the assembly with repeatmodeler 2.0.3 (Flynn et al. 2020). The classified de novo repeats were combined with mammal repeats from the Dfam and Repbase databases (downloaded 20170127) and annotated in the assembly using repeatmasker 4.0.7 Smit et al. (2013–2015). The final dataset contained 21.3 million SNPs.

To account for parts in the focal genes that were removed in manual editing of the cross-species alignments used to calculate dN/dS (see above), we mapped, for each gene, the trimmed CDS for the house mouse and bank vole back to their respective assembly. This was done using minimap2 v.2.26 (Li, 2018), specifying the option for spliced alignments from high-quality transcripts (-x splice:hq) together with splice junctions extracted from the annotation files and only reporting the best hit (-N 1). The alignments were converted into a bam format using samtools v1.20 (Li et al. 2009) and subsequently converted into a bed format using the bamToBed function in bedtools v2.31.1 (Quinlan and Hall 2010). Next, bedtools was used to intersect the aligned intervals with the CDS from the genome annotation for each assembly. Based on the intersection, we filtered the raw alignments to contain only those where the gene name was matching that of the overlapping genome annotation. In the bank vole, we also allowed matches to genes that we previously knew had a different name in the official annotations compared with the TOGA-derived annotations used for the aligned sequences. We also used bcftools to filter the variant (vcf) files of each species to only contain SNPs overlapping the filtered aligned intervals.

Degenotate was run with default settings except for specifying a minor allele frequency of 0.0625 in the bank vole, to account for the smaller sample size in the house mouse. We also added an artificial outgroup sample to each vcf file, which was homozygous for the reference allele (0/0), to get the number of synonymous and nonsynonymous SNPs for each transcript. We filtered the per-site codon degeneracy output file to only contain positions that were overlapping with the filtered alignments and summarized for each transcript the number of 0-fold (f0), 2-fold (f2), 3-fold (f3), and 4-fold degenerate sites (f4).

For the bank vole, we selected for each gene data from the NCBI transcript that had the largest intersection with the mouse-derived TOGA gene annotation. For the house mouse, we selected the same transcript as provided in the TOGA annotation or, if the specific transcript was not included, the longest one from the same gene. From the degenerate site counts, we estimated the number of synonymous (0 × f0 + 1/3 × f2 + 2/3 × f3 + 1 × f4) and nonsynonymous sites (1 × f0 + 2/3 × f2 + 1/3 × f3 + 0 × f4) for each transcript (Nei and Gojobori 1986). The number of synonymous and nonsynonymous variants was divided by the number of synonymous and nonsynonymous sites to obtain pS and pN, respectively. To avoid missing values for pN/pS when pS = 0, we added 1 to all values of S, that is, p(S + 1) (Ebel et al. 2017).

### Gene Expression and Other Covariates

Expression levels of immune and control genes were estimated from ten organs (brain, heart, kidney, liver, lung, ovary, pancreas, spleen, small intestine [ileum], and testis) of house mouse and bank vole.

For the house mouse, we used publicly available transcriptome data generated as a part of the ENCODE project (Dunham et al. 2012). In total, 25 samples across ten tissues from both sexes were downloaded from SRA (supplementary table S6, Supplementary Material online).

For the bank vole, we generated transcriptome data from ten tissues of wild-caught animals (*n* = 19), spanning both sexes and two seasons (summer and winter) to capture a broad transcriptome profile for each gene. Wild bank voles were captured using live traps during early September 2022 and January 2023 at Revingehed, 20 km east of Lund, Sweden. Trapped individuals were weighed, sexed, euthanized, and immediately dissected. Organ samples were harvested in RNAlater (Invitrogen, Thermo Fisher Scientific) and stored at −80 °C. RNA samples were extracted in four batches. Less than 30 mg of tissue frozen in RNAlater was transferred to tubes containing the lysis buffer RLT of the RNEasy Plus kit (Qiagen), 2 M DTT, and 1% v/v of the antifoaming agent Reagent DX (Qiagen). Tissues were repeatedly homogenized using a TissueLyser II (Qiagen) at 30 Hz for 60-s intervals until no visible tissue clumps were present. Samples were cooled on ice between each round of homogenization. The homogenized samples were centrifuged at maximum speed for 5 min to pellet cell debris, whereafter the RNEasy Plus protocol was followed. The RNA was eluted with RNAse free water, and the purity was checked with a NanoDrop (Thermo Fisher Scientific). The integrity of the extracted RNA was determined with BioAnalyser II (Agilent), and the concentration was estimated using RiboGreen assay kit (Thermo Fisher Scientific). Samples with sufficient concentration and RIN values > 7 had libraries prepared using a TruSeq Stranded mRNA (Illumina, USA) protocol and were sequenced on a NovaSeq6000 S4 flow cell (Illumina) using a 2 × 150 bp setup. The sequencing followed standard protocols except for using xGen UDI-UMI adapters (Integrated DNA Technologies, USA), which have dual indices that minimize index hopping and also result in the strandedness of the reads being flipped. All sequencing was performed by the NGI, Stockholm, Sweden.

Downstream analyses of the RNAseq data were performed similarly for both species. Raw reads were trimmed for adapter content and quality using Trimmomatic v0.39 (Bolger et al. 2014) with the settings ILLUMINACLIP: TruSeq3-PE-2.fa:2:30:10 LEADING:5 TRAILING:5 SLIDINGWINDOW:4:20 MINLEN:40. Reads were mapped against the respective reference genomes (GRCm39 for house mouse and GCF_902806735.1 for bank vole) using STAR v2.7.11a (Dobin et al. 2013). The annotation gtf file provided with each assembly (house mouse: Ensembl release 110; bank vole: NCBI release 100) was used in the construction of the star genome database, which allowed us to get alignments to the transcriptome (−quantMode TranscriptomeSAM). The transcriptome alignment files were used as input to RSEM v1.3.1 (Li and Dewey 2011), which was run with recommended settings for paired-end data and specifying the strandedness of the reads (forward) in the case of the bank vole.

To compare expression levels between the bank vole and mouse samples, we first identified a set of orthologous genes between the species. To this end, we compared projected mouse annotations from TOGA to the bank vole assembly and the NCBI annotation. In each of the two gtf files, we extracted CDS positions for each transcript and intersected the two datasets with bedtools 2.31.1 (Quinlan and Hall 2010). From the intersection, we summarized how many genes each NCBI gene and TOGA gene were overlapping. For the set of orthologs, we excluded cases where an NCBI gene was overlapping with multiple TOGA genes, where a TOGA gene was overlapping with multiple NCBI genes, where a TOGA gene had been assigned any form of orthology including the term “many” and TOGA genes with IDs that could not be matched to the gene annotation used for the mouse gene expression analysis. This resulted in a final list of 15,570 gene IDs that was used to filter the gene-level RSEM output from each bank vole and mouse sample.

Gene-level output from RSEM filtered for mouse–bank vole orthologs was imported into edgeR (Robinson et al. 2009) using tximport (Soneson et al. 2016). In edgeR, expected read counts were converted into counts per million (CPM) and adjusted for total library sizes using a trimmed mean of *M*-values (TMM [Robinson and Oshlack 2010]) method. The CPM values for each sample were divided by the (sample-specific) effective length of each gene to obtain TMM–FPKM values.

Tissue specificity (*τ*) (Yanai et al. 2005) was calculated based on mean TMM–FPKM values for each tissue. Genes where TMM–FPKM values were zero across all tissues (ten genes for house mouse and 23 for bank vole) were excluded.

To visualize overall expression differences among different tissues and between species, we used a PCA implemented in DESeq2 (Love et al. 2014). For this purpose, expected counts from the gene-level RSEM output for each sample were first imported with tximport. In the dataset, genes with a length of 0 were assigned a length of 0.01 to avoid errors, genes with too low expression were removed (requiring at least four reads and at least three samples), and the expected counts were normalized using a variance-stabilizing transformation before running the PCA. The results of the PCA were visualized in R using ggplot2.

PPI data from mice were obtained from the STRING database v12 (Szklarczyk et al. 2023). For each gene, the number of PPI with medium confidence (≥0.4) was obtained. Gene length was measured as the length of the multiple sequence alignment.

### Statistical Analyses

For statistical analyses, dN/dS was square root transformed to normalize the distribution of residuals. Mean expression, PPI, and sequence length were log_10_ transformed, and *τ* was arsin(sqrt) transformed. All continuous predictor variables (mean expression, *τ*, PPI, sequence length, pN/pS) were also *Z*-transformed.

Analyses were performed with R using the lm() function for dN/dS and the glm() function with family = binomial for positive selection (coded as 0 or 1). Statistical significance was assessed by type 3 F tests for dN/dS and LR tests for positive selection. Post hoc tests were performed as Dunnett's test (comparing each immune gene category against the control genes) using the emmeans package (Lenth 2024). pN/pS could not be transformed so as to not violate assumptions of parametric statistical tests, so a Kruskal–Wallis test was used instead, with Dunn's post hoc test (with the Benjamini–Hochberg correction for six comparisons, i.e. each immune gene category against controls, so as to correspond to the Dunnett's test used in other analyses). Figures were generated with the ggplot2 and visreg packages (Wickham 2016; Breheny and Burchett 2017).

## Supplementary Material

msaf016_Supplementary_Data

## Data Availability

Bank vole transcriptome data are available at SRA (PRJNA1119683). Datasets and code are available at GitHub (https://github.com/lraberg/PositiveSelectionRodentImmuneGenes).

## References

[msaf016-B1] Adrian J, Bonsignore P, Hammer S, Frickey T, Hauck CR. Adaptation to host-specific bacterial pathogens drives rapid evolution of a human innate immune receptor. Curr Biol. 2019:29(4):616–630. 10.1016/j.cub.2019.01.058. 30744974

[msaf016-B2] Agrawal A, Lively CM. Infection genetics: gene-for-gene versus matching-alleles models and all points in between. Evol Ecol Res. 2002:4:79–90.

[msaf016-B3] Alcaide M, Edwards SV. Molecular evolution of the toll-like receptor multigene family in birds. Mol Biol Evol. 2011:28(5):1703–1715. 10.1093/molbev/msq351. 21239391

[msaf016-B4] Aleksander SA, Balhoff J, Carbon S, Cherry JM, Drabkin HJ, Ebert D, Feuermann M, Gaudet P, Harris NL, Hill DP, et al The gene ontology knowledgebase in 2023. Genetics. 2023:224(1):iyad031. 10.1093/genetics/iyad031. PMC10158837 36866529

[msaf016-B5] Álvarez-Carretero S, Kapli P, Yang Z. Beginner's guide on the use of PAML to detect positive selection. Mol Biol Evol. 2023:40(4):1–18. 10.1093/molbev/msad041. PMC10127084 37096789

[msaf016-B6] Babik W, Dudek K, Fijarczyk A, Pabijan M, Stuglik M, Szkotak R, Zieliński P. Constraint and adaptation in newt toll-like receptor genes. Genome Biol Evol. 2014:7(1):81–95. 10.1093/gbe/evu266. 25480684 PMC4316619

[msaf016-B7] Benjamini Y, Hochberg Y. Controlling the false discovery rate: a practical and powerful approach to multiple testing. J R Stat Soc Ser B. 1995:57(1):289–300. 10.1111/j.2517-6161.1995.tb02031.x.

[msaf016-B8] Beyer DK, Forero A. Mechanisms of antiviral immune evasion of SARS-CoV-2. J Mol Biol. 2022:434(6):167265. 10.1016/j.jmb.2021.167265. 34562466 PMC8457632

[msaf016-B9] Bolger AM, Lohse M, Usadel B. Trimmomatic: a flexible trimmer for Illumina sequence data. Bioinformatics. 2014:30(15):2114–2120. 10.1093/bioinformatics/btu170. 24695404 PMC4103590

[msaf016-B10] Brawand D, Soumillon M, Necsulea A, Julien P, Csárdi G, Harrigan P, Weier M, Liechti A, Aximu-Petri A, Kircher M, et al The evolution of gene expression levels in mammalian organs. Nature. 2011:478(7369):343–348. 10.1038/nature10532. 22012392

[msaf016-B11] Breheny P, Burchett W. Visualization of regression models using visreg. R J. 2017:9(2):56–71. 10.32614/RJ-2017-046.

[msaf016-B12] Cagliani R, Forni D, Filippi G, Mozzi A, De Gioia L, Pontremoli C, Pozzoli U, Bresolin N, Clerici M, Sironi M. The mammalian complement system as an epitome of host-pathogen genetic conflicts. Mol Ecol. 2016:25(6):1324–1339. 10.1111/mec.13558. 26836579

[msaf016-B13] Danecek P, Auton A, Abecasis G, Albers CA, Banks E, Depristo MA, Handsaker RE, Lunter G, Marth GT, Sherry ST, et al The variant call format and VCFtools. Bioinformatics. 2011:27(15):2156–2158. 10.1093/bioinformatics/btr330. 21653522 PMC3137218

[msaf016-B14] Danecek P, Bonfield JK, Liddle J, Marshall J, Ohan V, Pollard MO, Whitwham A, Keane T, McCarthy SA, Davies RM, et al Twelve years of SAMtools and BCFtools. Gigascience. 2021:10(2):giab008. 10.1093/gigascience/giab008. PMC7931819 33590861

[msaf016-B15] Demeure CE, Dussurget O, Mas Fiol G, Le Guern A-S, Savin C, Pizarro-Cerdá J. Yersinia pestis and plague: an updated view on evolution, virulence determinants, immune subversion, vaccination, and diagnostics. Genes Immun. 2019:20(5):357–370. 10.1038/s41435-019-0065-0. 30940874 PMC6760536

[msaf016-B16] Deschamps M, Laval G, Fagny M, Itan Y, Abel L, Casanova J-L. Genomic signatures of selective pressures and introgression from archaic hominins at human innate immunity genes. Am J Hum Genet. 2016:98(1):5–21. 10.1016/j.ajhg.2015.11.014. 26748513 PMC4716683

[msaf016-B17] Dobin A, Davis CA, Schlesinger F, Drenkow J, Zaleski C, Jha S, Batut P, Chaisson M, Gingeras TR. STAR: ultrafast universal RNA-seq aligner. Bioinformatics. 2013:29(1):15–21. 10.1093/bioinformatics/bts635. 23104886 PMC3530905

[msaf016-B18] Dunham I, Kundaje A, Aldred SF, Collins PJ, Davis CA, Doyle F. An integrated encyclopedia of DNA elements in the human genome. Nature. 2012:489(7414):57–74. 10.1038/nature11247. 22955616 PMC3439153

[msaf016-B19] Durinck S, Spellman PT, Birney E, Huber W. Mapping identifiers for the integration of genomic datasets with the R/Bioconductor package biomaRt. Nat Protoc. 2009:4(8):1184–1191. 10.1038/nprot.2009.97. 19617889 PMC3159387

[msaf016-B20] Dybdahl MF, Jenkins CE, Nuismer SL. Identifying the molecular basis of host-parasite coevolution: merging models and mechanisms. Am Nat. 2014:184(1):1–13. 10.1086/676591. 24921596

[msaf016-B21] Ebel ER, Telis N, Venkataram S, Petrov DA, Enard D. High rate of adaptation of mammalian proteins that interact with Plasmodium and related parasites. PLoS Genet. 2017:13(9):1–27. 10.1371/journal.pgen.1007023. PMC5634635 28957326

[msaf016-B22] Ebert D, Fields PD. Host–parasite co-evolution and its genomic signature. Nat Rev Genet. 2020:21(12):754–768. 10.1038/s41576-020-0269-1. 32860017

[msaf016-B23] Enard D, Cai L, Gwennap C, Petrov DA. Viruses are a dominant driver of protein adaptation in mammals. Elife. 2016:5:e12469. 10.7554/eLife.12469. 27187613 PMC4869911

[msaf016-B24] Fabre P-H, Hautier L, Dimitrov D, Douzery EJP. A glimpse on the pattern of rodent diversification: a phylogenetic approach. BMC Evol Biol. 2012:12(1):88. 10.1186/1471-2148-12-88. 22697210 PMC3532383

[msaf016-B25] Fabregat A, Sidiropoulos K, Viteri G, Marin-Garcia P, Ping P, Stein L, D’Eustachio P, Hermjakob H. Reactome diagram viewer: data structures and strategies to boost performance. Bioinformatics. 2018:34(7):1208–1214. 10.1093/bioinformatics/btx752. 29186351 PMC6030826

[msaf016-B26] Flynn JM, Hubley R, Goubert C, Rosen J, Clark AG, Feschotte C, Smit AF. RepeatModeler2 for automated genomic discovery of transposable element families. Proc Natl Acad Sci U S A. 2020:117(17):9451–9457. 10.1073/pnas.1921046117. 32300014 PMC7196820

[msaf016-B27] Garrison E, Kronenberg ZN, Dawson ET, Pedersen BS, Prins P. A spectrum of free software tools for processing the VCF variant call format: vcflib, bio-vcf, cyvcf2, hts-nim and slivar. PLoS Comput Biol. 2022:18(5):e1009123. 10.1371/journal.pcbi.1009123. 35639788 PMC9286226

[msaf016-B28] Garrison E, Marth, G. Haplotype-based variant detection from short-read sequencing. arXiv 1207.3907v. 10.48550/arXiv.1207.3907, 20 July 2012, preprint: not peer reviewed.

[msaf016-B29] Hagai T, Chen X, Miragaia RJ, Rostom R, Gomes T, Kunowska N, Henriksson J, Park J-E, Proserpio V, Donati G, et al Gene expression variability across cells and species shapes innate immunity. Nature. 2018:563(7730):197–202. 10.1038/s41586-018-0657-2. 30356220 PMC6347972

[msaf016-B30] Harr B, Karakoc E, Neme R, Teschke M, Pfeifle C, Pezer Ž, Babiker H, Linnenbrink M, Montero I, Scavetta R, et al Genomic resources for wild populations of the house mouse, *Mus musculus* and its close relative *Mus spretus*. Sci Data. 2016:3(1):160075. 10.1038/sdata.2016.75. 27622383 PMC5020872

[msaf016-B31] Hoang DT, Chernomor O, Von Haeseler A, Minh BQ, Vinh LS. UFBoot2: improving the ultrafast bootstrap approximation. Mol Biol Evol. 2018:35(2):518–522. 10.1093/molbev/msx281. 29077904 PMC5850222

[msaf016-B32] Janeway CA . Approaching the asymptote? Evolution and revolution in immunology. Cold Spring Harb Symp Quant Biol. 1989:54(0):1–13. 10.1101/SQB.1989.054.01.003. 2700931

[msaf016-B33] Kalyaanamoorthy S, Minh BQ, Wong TKF, Von Haeseler A, Jermiin LS. ModelFinder: fast model selection for accurate phylogenetic estimates. Nat Methods. 2017:14(6):587–589. 10.1038/nmeth.4285. 28481363 PMC5453245

[msaf016-B34] Kanehisa M, Furumichi M, Tanabe M, Sato Y, Morishima K. KEGG: new perspectives on genomes, pathways, diseases and drugs. Nucleic Acids Res. 2017:45(D1):353–361. 10.1093/nar/gkw1092. 27899662 PMC5210567

[msaf016-B35] Kirilenko BM, Munegowda C, Osipova E, Jebb D, Sharma V, Blumer M, Morales AE, Ahmed A-W, Kontopoulos D-G, Hilgers L, et al Integrating gene annotation with orthology inference at scale. Science. 2023:380(6643):368. 10.1126/science.abn3107. PMC10193443 37104600

[msaf016-B36] Kosakovsky Pond SL, Poon AFY, Velazquez R, Weaver S, Hepler NL, Murrell B, Shank SD, Magalis BR, Bouvier D, Nekrutenko A, et al Hyphy 2.5—a customizable platform for evolutionary hypothesis testing using phylogenies. Mol Biol Evol. 2020:37(1):295–299. 10.1093/molbev/msz197. 31504749 PMC8204705

[msaf016-B37] Kosiol C, Holmes I, Goldman N. An empirical codon model for protein sequence evolution. Mol Biol Evol. 2007:24(7):1464–1479. 10.1093/molbev/msm064. 17400572

[msaf016-B38] Kosiol C, Vinar T, Fonseca RR, Hubisz MJ, Bustamante CD, Nielsen R, Siepel A. Patterns of positive selection in six mammalian genomes. PLoS Genet. 2008:4(8):e1000144. 10.1371/journal.pgen.1000144. 18670650 PMC2483296

[msaf016-B39] Lenth RV . 2024. emmeans: estimated marginal means, aka least-squares means. R package version 1.10. https://rvlenth.github.io/emmeans/.

[msaf016-B40] Li B, Dewey CN. RSEM: accurate transcript quantification from RNA-Seq data with or without a reference genome. BMC Bioinformatics. 2011:12(1):323. 10.1186/1471-2105-12-323. 21816040 PMC3163565

[msaf016-B41] Li H . Minimap2: pairwise alignment for nucleotide sequences. Bioinformatics. 2018:34(18):3094–3100. 10.1093/bioinformatics/bty191. 29750242 PMC6137996

[msaf016-B42] Li H, Handsaker B, Wysoker A, Fennell T, Ruan J, Homer N, Marth G, Abecasis G, Durbin R. The sequence alignment/map format and SAMtools. Bioinformatics. 2009:25(16):2078–2079. 10.1093/bioinformatics/btp352. 19505943 PMC2723002

[msaf016-B43] Love MI, Huber W, Anders S. Moderated estimation of fold change and dispersion for RNA-Seq data with DESeq2. Genome Biol. 2014:15(12):550. 10.1186/s13059-014-0550-8. 25516281 PMC4302049

[msaf016-B44] Löytynoja A, Goldman N. Phylogeny-aware gap placement prevents errors in sequence alignment and evolutionary analysis. Science. 2008:320(5883):1632–1635. 10.1126/science.1158395. 18566285

[msaf016-B45] Lucaci AG, Zehr JD, Enard D, Thornton JW, Kosakovsky Pond SL. Evolutionary shortcuts via multinucleotide substitutions and their impact on natural selection analyses. Mol Biol Evol. 2023:40(7):msad150. 10.1093/molbev/msad150. PMC10336034 37395787

[msaf016-B46] Lundberg M, Zhong X, Konrad A, Olsen R-A, Råberg L. Balancing selection in pattern recognition receptor pathways is associated with pleiotropy and gene function. Mol Ecol. 2020:29(11):1990–2003. 10.1111/mec.15459. 32374503

[msaf016-B47] Mi H, Muruganujan A, Huang X, Ebert D, Mills C, Guo X, Thomas PD. Protocol update for large-scale genome and gene function analysis with the PANTHER classification system (v.14.0). Nat Protoc. 2019:14(3):703–721. 10.1038/s41596-019-0128-8. 30804569 PMC6519457

[msaf016-B48] Minh BQ, Schmidt HA, Chernomor O, Schrempf D, Woodhams MD, Von Haeseler A, Lanfear R. IQ-TREE 2: new models and efficient methods for phylogenetic inference in the genomic era. Mol Biol Evol. 2020:37(5):1530–1534. 10.1093/molbev/msaa015. 32011700 PMC7182206

[msaf016-B49] Mirchandani CD, Shultz AJ, Thomas GWC, Smith SJ, Baylis M, Arnold B, Corbett-Detig R, Enbody E, Sackton TB. A fast, reproducible, high-throughput variant calling workflow for population genomics. Mol Biol Evol. 2024:41(1):msad270. 10.1093/molbev/msad270. PMC10764099 38069903

[msaf016-B50] Murphy K, Weaver CT, Berg L. Janeway's immunobiology. 10th ed. London: Garland Science; 2022.

[msaf016-B51] Murrell B, Weaver S, Smith MD, Wertheim JO, Murrell S, Aylward A, Eren K, Pollner T, Martin DP, Smith DM, et al Gene-wide identification of episodic selection. Mol Biol Evol. 2015:32(5):1365–1371. 10.1093/molbev/msv035. 25701167 PMC4408417

[msaf016-B52] Murrell B, Wertheim JO, Moola S, Weighill T, Scheffler K, Kosakovsky Pond SL. Detecting individual sites subject to episodic diversifying selection. PLoS Genet. 2012:8(7):e1002764. 10.1371/journal.pgen.1002764. 22807683 PMC3395634

[msaf016-B53] Nei M, Gojobori T. Simple methods for estimating the numbers of synonymous and nonsynonymous nucleotide substitutions. Mol Biol Evol. 1986:3(5):418–426. 10.1093/oxfordjournals.molbev.a040410. 3444411

[msaf016-B54] Obbard DJ, Welch JJ, Kim K-W, Jiggins FM. Quantifying adaptive evolution in the *Drosophila* immune system. PLoS Genet. 2009:5(10):e1000698. 10.1371/journal.pgen.1000698. 19851448 PMC2759075

[msaf016-B55] Quinlan AR, Hall IM. BEDTools: a flexible suite of utilities for comparing genomic features. Bioinformatics. 2010:26(6):841–842. 10.1093/bioinformatics/btq033. 20110278 PMC2832824

[msaf016-B56] Råberg L . Human and pathogen genotype-by-genotype interactions in the light of coevolution theory. PLoS Genet. 2023:19(4):e1010685. 10.1371/journal.pgen.1010685. 37023017 PMC10079023

[msaf016-B57] RCoreTeam . R: a language and environment for statistical computing. Vienna, Austria: R Foundation for Statistical Computing; 2022.

[msaf016-B58] Robinson MD, McCarthy DJ, Smyth GK. Edger: a Bioconductor package for differential expression analysis of digital gene expression data. Bioinformatics. 2009:26(1):139–140. 10.1093/bioinformatics/btp616. 19910308 PMC2796818

[msaf016-B59] Robinson MD, Oshlack A. A scaling normalization method for differential expression analysis of RNA-seq data. Genome Biol. 2010:11(3):R25. 10.1186/gb-2010-11-3-r25. 20196867 PMC2864565

[msaf016-B60] Roycroft E, Achmadi A, Callahan CM, Esselstyn JA, Good JM, Moussalli A, Rowe KC. Molecular evolution of ecological specialisation: genomic insights from the diversification of murine rodents. Genome Biol Evol. 2021:13(7):evab103. 10.1093/gbe/evab103. 33988699 PMC8258016

[msaf016-B61] Sackton TB, Lazzaro BP, Schlenke TA, Evans JD, Hultmark D, Clark AG. Dynamic evolution of the innate immune system in *Drosophila*. Nat Genet. 2007:39(12):1461–1468. 10.1038/ng.2007.60. 17987029

[msaf016-B62] Schloissnig S, Arumugam M, Sunagawa S, Mitreva M, Tap J, Zhu A, Waller A, Mende DR, Kultima JR, Martin J, et al Genomic variation landscape of the human gut microbiome. Nature. 2013:493(7430):45–50. 10.1038/nature11711. 23222524 PMC3536929

[msaf016-B63] Shen W-K, Chen S-Y, Gan Z-Q, Zhang Y-Z, Yue T, Chen M-M, Xue Y, Hu H, Guo A-Y. AnimalTFDB 4.0: a comprehensive animal transcription factor database updated with variation and expression annotations. Nucleic Acids Res. 2023:51(D1):D39–D45. 10.1093/nar/gkac907. 36268869 PMC9825474

[msaf016-B64] Shultz AJ, Sackton TB. Immune genes are hotspots of shared positive selection across birds and mammals. Elife. 2019:8:e41815. 10.7554/eLife.41815. 30620335 PMC6338464

[msaf016-B65] Smit AFA, Hubley R, Green P. RepeatMasker Open. 4.0. 2013–2015. http://www.repeatmasker.org.

[msaf016-B66] Soneson C, Love MI, Robinson MD. Differential analyses for RNA-seq: transcript-level estimates improve gene-level inferences. F1000Res. 2016:4:1521. 10.12688/f1000research.7563.2. PMC4712774 26925227

[msaf016-B67] Soni V, Eyre-Walker A. Factors that affect the rates of adaptive and nonadaptive evolution at the gene level in humans and chimpanzees. Genome Biol Evol. 2022:14(2):evac028. 10.1093/gbe/evac028. 35166775 PMC8882387

[msaf016-B68] Souilmi Y, Lauterbur ME, Tobler R, Huber CD, Johar AS, Moradi SV, Johnston WA, Krogan NJ, Alexandrov K, Enard D. An ancient viral epidemic involving host coronavirus interacting genes more than 20,000 years ago in East Asia. Curr Biol. 2021:31(16):3504–3514.e9. 10.1016/j.cub.2021.05.067. 34171302 PMC8223470

[msaf016-B69] Szklarczyk D, Kirsch R, Koutrouli M, Nastou K, Mehryary F, Hachilif R, Gable AL, Fang T, Doncheva NT, Pyysalo S. The STRING database in 2023: protein–protein association networks and functional enrichment analyses for any sequenced genome of interest. Nucleic Acids Res. 2023:51(D1):D638–D646. 10.1093/nar/gkac1000. 36370105 PMC9825434

[msaf016-B70] Tange O . 2022. GNU Parallel. 20220422. Zenodo. 10.5281/zenodo.11043435.

[msaf016-B71] Tehthorey JL, Haloupek N, López-blanco JR, Grob P, Adamson E, Hartenian E, Lind NA, Bourgeois NM, Chacón P, Nogales E, et al The structural basis of flagellin detection by NAIP5: a strategy to limit pathogen immune evasion. Science. 2017:893(6365):888–893. 10.1126/science.aao1140. PMC5842810 29146805

[msaf016-B72] The UniProt Consortium . UniProt: the universal protein knowledgebase. Nucleic Acids Res. 2017:45(D1):D158–D169. 10.1093/nar/gkw1099. 27899622 PMC5210571

[msaf016-B73] Turner AK, Begon M, Jackson JA, Paterson S. Evidence for selection at cytokine loci in a natural population of field voles (*Microtus agrestis*). Mol Ecol. 2012:21(7):1632–1646. 10.1111/j.1365-294X.2012.05501.x. 22364125

[msaf016-B74] Velová H, Gutowska-ding MW, Burt DW, Vinkler M. Toll-like receptor evolution in birds: gene duplication, pseudogenization, and diversifying selection. Mol Biol Evol. 2018:35(9):2170–2184. 10.1093/molbev/msy119. 29893911 PMC6107061

[msaf016-B75] Venkat A, Hahn MW, Thornton JW. Multinucleotide mutations cause false inferences of lineage-specific positive selection. Nat Ecol Evol. 2018:2(8):1280–1288. 10.1038/s41559-018-0584-5. 29967485 PMC6093625

[msaf016-B76] Vinkler M, Albrecht T. The question waiting to be asked: innate immunity receptors in the perspective of zoological research. Folia Zool. 2009:58:15–28.

[msaf016-B77] Vinkler M, Fiddaman SR, Těšický M, O’Connor EA, Savage AE, Lenz TL, Smith AL, Kaufman J, Bolnick DI, Davies CS, et al Understanding the evolution of immune genes in jawed vertebrates. J Evol Biol. 2023:36(6):847–873. 10.1111/jeb.14181. 37255207 PMC10247546

[msaf016-B78] Wang G, Li X, Wang Z. APD3: the antimicrobial peptide database as a tool for research and education. Nucleic Acids Res. 2016:44(D1):D1087–D1093. 10.1093/nar/gkv1278. 26602694 PMC4702905

[msaf016-B79] Wickham H . Ggplot2: elegant graphics for data analysis. New York: Springer-Verlag; 2016.

[msaf016-B80] Wisotsky SR, Kosakovsky Pond SL, Shank SD, Muse SV. Synonymous site-to-site substitution rate variation dramatically inflates false positive rates of selection analyses: ignore at your own peril. Mol Biol Evol. 2021:37(8):2430–2439. 10.1093/molbev/msaa037. PMC7403620 32068869

[msaf016-B81] Wlasiuk G, Nachman MW. Adaptation and constraint at Toll-like receptors in primates. Mol Biol Evol. 2010:27(9):2172–2186. 10.1093/molbev/msq104. 20410160 PMC3107592

[msaf016-B82] Yanai I, Benjamin H, Shmoish M, Chalifa-Caspi V, Shklar M, Ophir R, Bar-Even A, Horn-Saban S, Safran M, Domany E, et al Genome-wide midrange transcription profiles reveal expression level relationships in human tissue specification. Bioinformatics. 2005:21(5):650–659. 10.1093/bioinformatics/bti042. 15388519

[msaf016-B83] Yang Z . PAML 4: phylogenetic analysis by maximum likelihood. Mol Biol Evol. 2007:24(8):1586–1591. 10.1093/molbev/msm088. 17483113

[msaf016-B84] Zhang J, Yang J-R. Determinants of the rate of protein sequence evolution. Nat Rev Genet. 2015:16(7):409–420. 10.1038/nrg3950. 26055156 PMC4523088

