# Supplementary material for: Positive Selection on Mammalian Immune Genes—Effects of Gene Function and Selective Constraint
Source: Mol Biol Evol. 2025 Jan 21;42(1):msaf016. doi: 10.1093/molbev/msaf016 (PMC11783303; doi:10.1093/molbev/msaf016)
Supplement: msaf016_Supplementary_Data [file msaf016_supplementary_data.pdf]

Positive selection on mammalian immune genes—effects of gene function and selective constraint

Mridula Nandakumar, Max Lundberg, Fredric Carlsson, Lars Råberg*

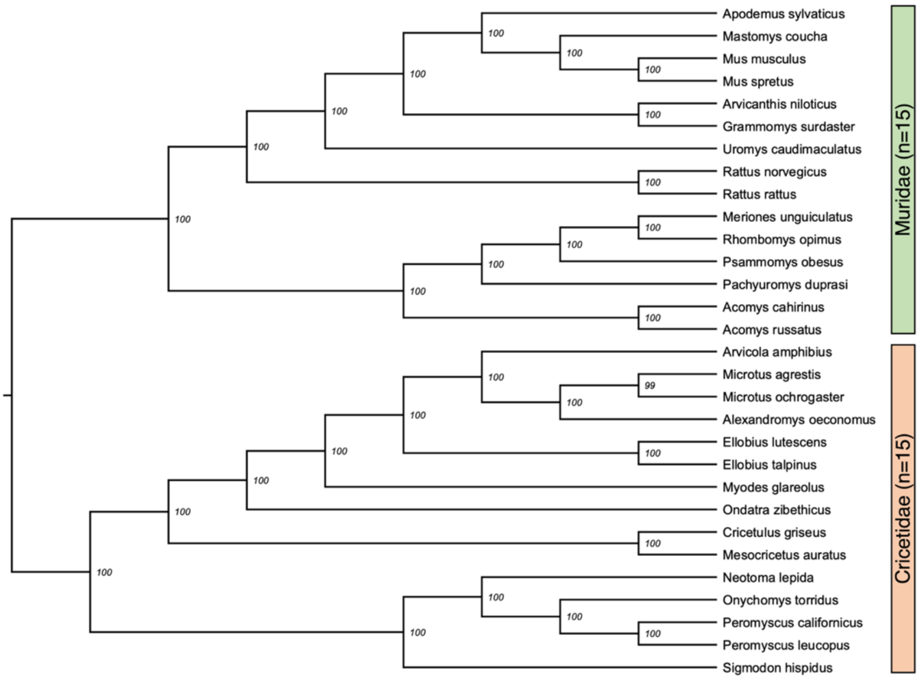

Figure S1. Phylogenetic tree for the rodent species included in the analyses of selection. Concatenated sequences from both immune and control gene sets were used for tree construction in IQTREE2. Confidence values were generated using Ultrafast Bootstrapping.

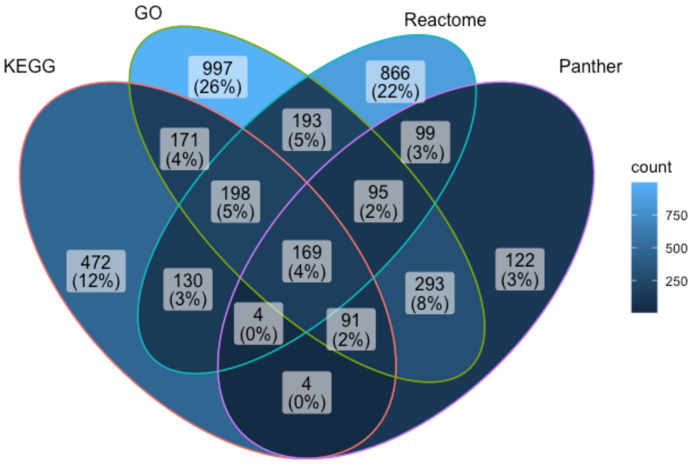

Figure S2. Venn diagram of immune genes from KEGG, AmiGO2, Reactome, and Panther.

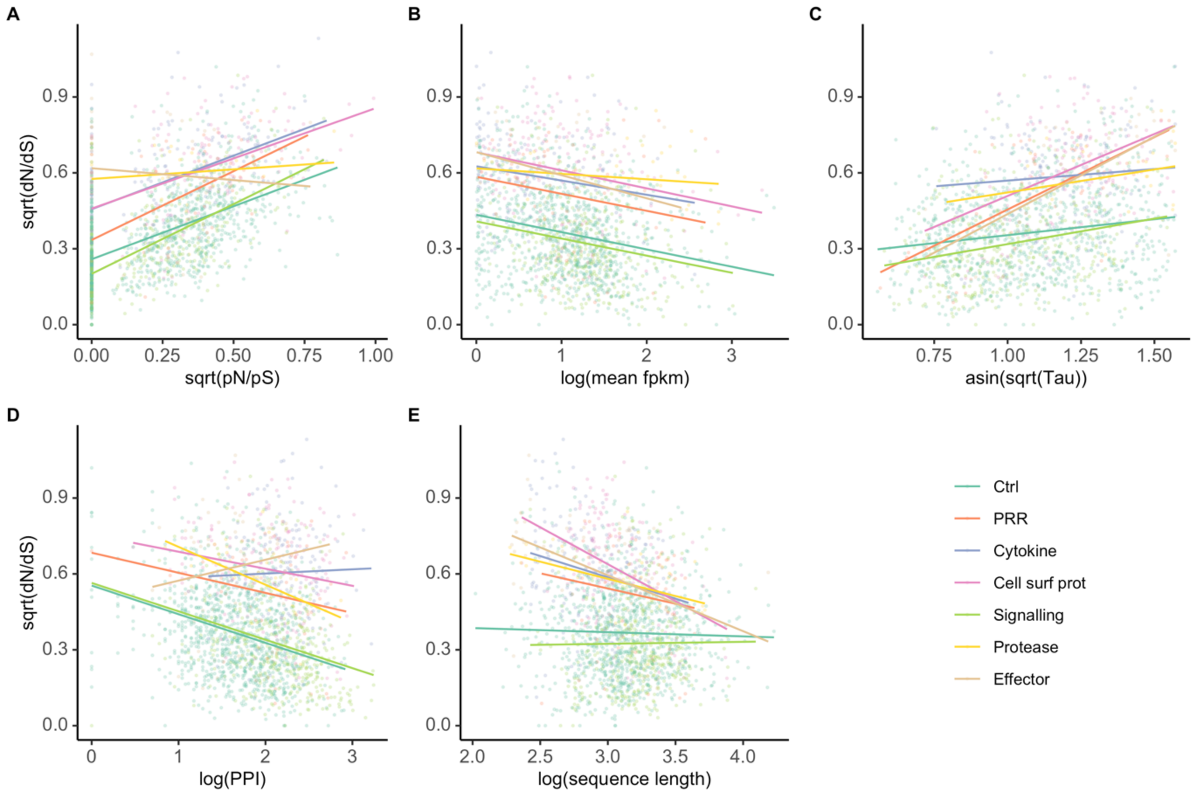

Figure S3. dN/dS (squareroot transformed) for six different categories of immune genes and non-immune control genes against A) pN/pS, B) mean gene expression, C) tissue specificity of expression, D) number of protein-protein interactions, and E) sequence length. For statistics, see table S5 and S6.

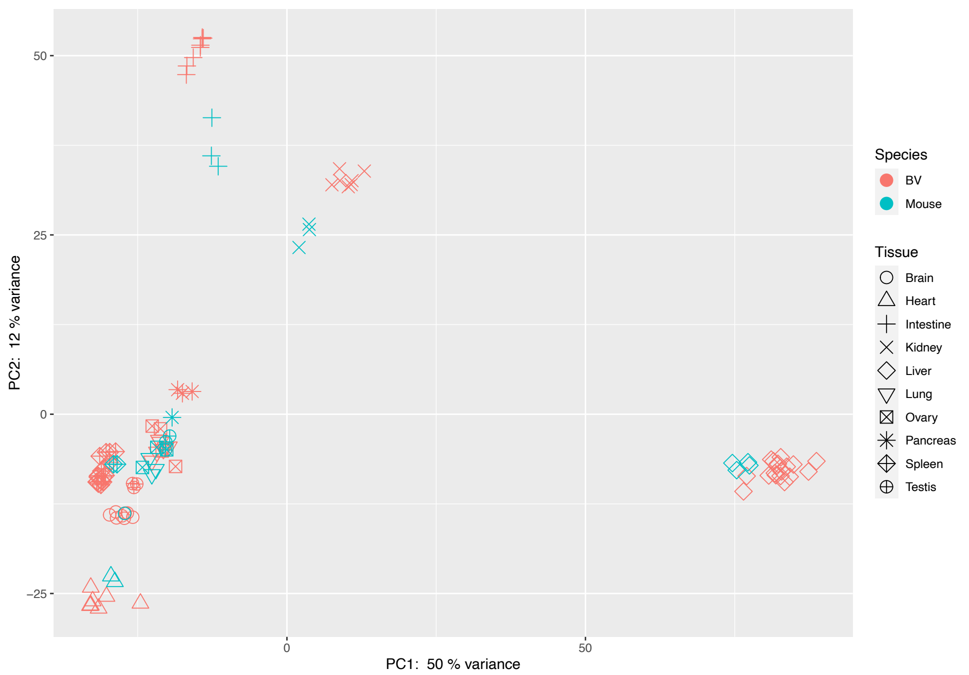

Figure S4. PCA based on expression of 15570 orthologs in ten tissues in house mouse and bank vole.

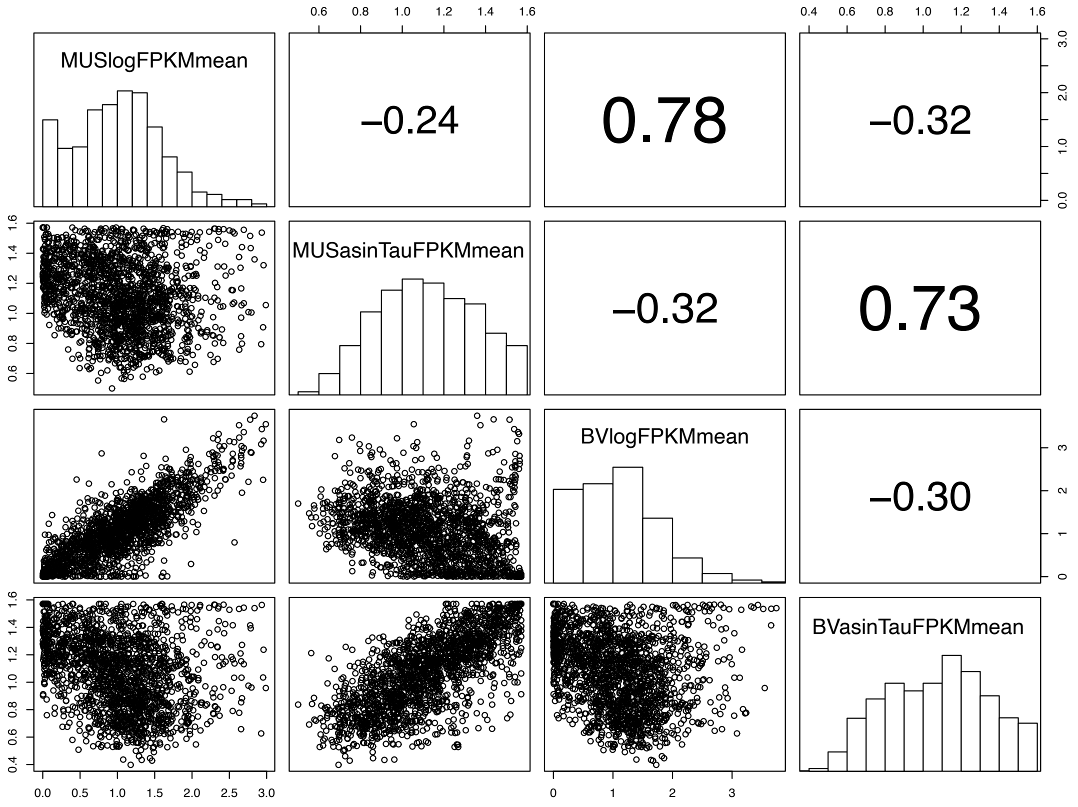

Figure S5. Correlations between mean expression in house mouse (MUSlogFPKMmean), mean expression in bank vole (BVlogFPKMmean), tissue specificity of expression in house mouse (MUSasinTauFPKMmean) and tissue specificity in bank vole (BVasinTauFPKMmean) for immune and control genes.

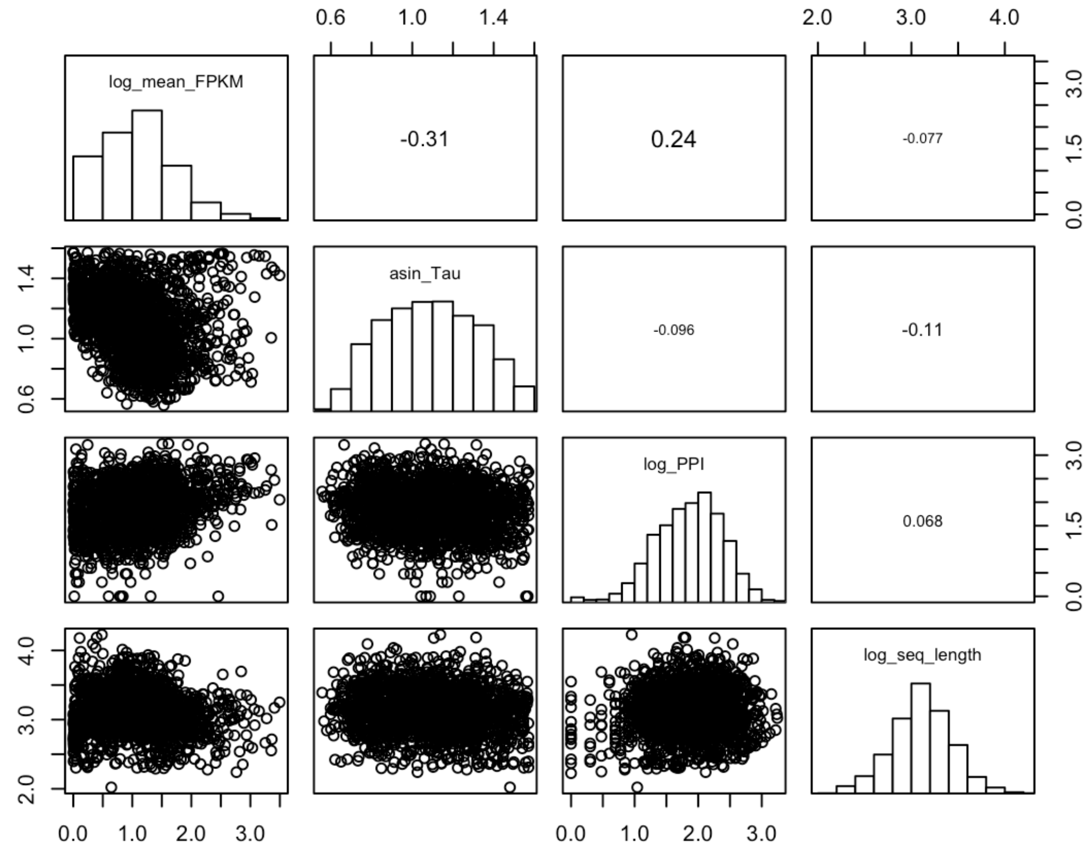

Figure S6: Correlations between mean expression (log transformed FPKM), tissue specificity of expression (asin(sqrt(Tau)), PPI (log transformed), and sequence length (log transformed) for immune and non-immune control genes.

Table S1 is available at https://github.com/lraberg/PositiveSelectionRodentImmuneGenes

Table S2: Immune genes with signatures of positive selection.

Gene	Gene Stable ID*	Category
App	ENSMUSG00000022892	Other cell surface proteins
Btk	ENSMUSG00000031264	Intracell. signalling proteins
C3ar1	ENSMUSG00000040552	Cyto/chemokines and their receptors
C8b	ENSMUSG00000029656	Extracell. proteases and inhibitors
Ccl26	ENSMUSG00000070464	Cyto/chemokines and their receptors
Cd244a	ENSMUSG00000004709	Other cell surface proteins
Cd48	ENSMUSG00000015355	Other cell surface proteins
Cd7	ENSMUSG00000025163	Other cell surface proteins
Cd80	ENSMUSG00000075122	Other cell surface proteins
Cd84	ENSMUSG00000038147	Other cell surface proteins
Ceacam20	ENSMUSG00000070777	Other cell surface proteins
Cfp	ENSMUSG00000001128	
Col3a1	ENSMUSG00000026043	
Cr1l	ENSMUSG00000016481	Other cell surface proteins
Cxcl16	ENSMUSG00000018920	Cyto/chemokines and their receptors
Cxcl9	ENSMUSG00000029417	Cyto/chemokines and their receptors
Defb18	ENSMUSG00000073735	Effector
Ifih1	ENSMUSG00000026896	PRR
Ifnb1	ENSMUSG00000048806	Cyto/chemokines and their receptors
Ifngr2	ENSMUSG00000022965	Cyto/chemokines and their receptors
Ikbkg	ENSMUSG00000004221	Intracell. signalling proteins
Il12b	ENSMUSG00000004296	Cyto/chemokines and their receptors
Il12rb1	ENSMUSG00000000791	Cyto/chemokines and their receptors
Il3	ENSMUSG00000018914	Cyto/chemokines and their receptors
Mapk9	ENSMUSG00000020366	Intracell. signalling proteins
Mill1	ENSMUSG00000054005	Other cell surface proteins
N4bp1	ENSMUSG00000031652	Effector
Ncf1	ENSMUSG00000015950	Intracell. signalling proteins
Nos3	ENSMUSG00000028978	Intracell. signalling proteins
Smarca4	ENSMUSG00000032187	Intracell. signalling proteins
Tet2	ENSMUSG00000040943	Intracell. signalling proteins
Tlr1	ENSMUSG00000044827	PRR
Tnfrsf11a	ENSMUSG00000026321	Cyto/chemokines and their receptors
Tnfsf8	ENSMUSG00000028362	Cyto/chemokines and their receptors
Trim26	ENSMUSG00000024457	Intracell. signalling proteins
Zbp1	ENSMUSG00000027514	PRR
Zbtb32	ENSMUSG00000006310	Intracell. signalling proteins

*ENSEMBL gene stable ID for house mouse gene

Table S3. General linear model of sqrt(dN/dS) against gene category, pN/pS, and their interaction. P-values from type III tests.

	df	F	P
Category	6	81.5	< 2.2e-16
Zsqrt(pN/pS)	1	294.1	< 2.2e-16
Category × Zsqrt(pN/pS)	6	5.3	1.88e-05
Residuals	1582		

All gene categories except signalling proteins had higher dN/dS than control genes (Dunnett's: P<0.0001).

Table S4. General linear model of sqrt(dN/dS) against gene category, mean expression, PPI, Tau, sequence length, and 2-way interaction between gene category and the covariates. Non-significant terms deleted at P>0.05 (interactions first). P-values from type III tests.

	df	F	P
Category	6	37.2	< 2.2e-16
Zlog(fpkm)	1	31.0	3.0e-08
Zlog(PPI)	1	57.9	4.9e-14
Zasin(sqrt(Tau))	1	5.6	0.018
Zlog(seq. length)	1	0.03	0.86
Category × Zlog(PPI)	6	4.0	0.0005
Category × Zasin(sqrt(Tau))	6	6.5	9.5e-07
Category × Zlog(seq. length))	6	3.2	0.0036
Residuals	1543		

Table S5: Details of public rodent genomes used for selection analysis

**Assembly Accession**	**Species**	**Family**
GCA_007455595.1	*Alexandromys oeconomus*	Cricetidae
GCA_903992535.2	*Arvicola amphibius*	Cricetidae
GCA_000223135.1	*Cricetulus griseus*	Cricetidae
GCA_001685075.1	*Ellobius lutescens*	Cricetidae
GCA_001685095.1	*Ellobius talpinus*	Cricetidae
GCA_017639785.1	*Mesocricetus auratus*	Cricetidae
GCA_902806775.1	*Microtus agrestis*	Cricetidae
GCA_000317375.1	*Microtus ochrogaster*	Cricetidae
GCA_902806735.1	*Myodes glareolus*	Cricetidae
GCA_001675575.1	*Neotoma lepida*	Cricetidae
GCA_004026605.1	*Ondatra zibethicus*	Cricetidae
GCA_903995425.1	*Onychomys torridus*	Cricetidae
GCA_007827085.3	*Peromyscus californicus*	Cricetidae
GCA_004664715.2	*Peromyscus leucopus*	Cricetidae
GCA_004025045.1	*Sigmodon hispidus*	Cricetidae
GCA_029890205.1	*Acomys cahirinus*	Muridae
GCA_903995435.1	*Acomys russatus*	Muridae
GCA_947179515.1	*Apodemus sylvaticus*	Muridae
GCA_011762505.1	*Arvicanthis niloticus*	Muridae
GCA_004785775.1	*Grammomys surdaster*	Muridae
GCA_008632895.1	*Mastomys coucha*	Muridae
GCA_002204375.1	*Meriones unguiculatus*	Muridae
GCA_000001635.9	*Mus musculus*	Muridae
GCA_921997135.2	*Mus spretus*	Muridae
GCA_028658305.1	*Pachyuromys duprasi*	Muridae
GCA_907164565.1	*Psammomys obesus*	Muridae
GCA_011064425.1	*Rattus rattus*	Muridae
GCA_015227675.2	*Rattus norvegicus*	Muridae
GCA_010120015.1	*Rhombomys opimus*	Muridae
GCA_028551405.1	*Uromys caudimaculatus*	Muridae

Table S6: Details of RNA samples for house mouse gene expression

SRA ID	ENCODE ID	Tissue
SRR5171102	ENCBS693QXI	brain
SRR5047921	ENCBS267ENC	heart
SRR5047924	ENCBS268ENC	heart
SRR5171094	ENCBS618GAD	kidney
SRR5047925	ENCBS270ENC	kidney
SRR5047928	ENCBS269ENC	kidney
SRR5171078	ENCBS391ULH	liver
SRR3192469	ENCBS093RNA	liver
SRR5047931	ENCBS279ENC	liver
SRR5047936	ENCBS278ENC	liver
SRR5171113	ENCBS964PGO	lung
SRR5047938	ENCBS292ENC	lung
SRR5047939	ENCBS291ENC	lung
SRR5171100	ENCBS887UIO	ovary
SRR5047985	ENCBS296ENC	ovary
SRR5047990	ENCBS295ENC	ovary
SRR5171086	ENCBS508NKR	pancreas
SRR5171081	ENCBS443BDC	small intestine
SRR5048001	ENCBS297ENC	small intestine
SRR5048006	ENCBS298ENC	small intestine
SRR5047941	ENCBS299ENC	spleen
SRR5047946	ENCBS300ENC	spleen
SRR5171084	ENCBS928FOT	testis
SRR5047954	ENCBS306ENC	testis
SRR5047955	ENCBS307ENC	testis
